# Impaired verbal memory function is related to anterior cingulate glutamate levels in schizophrenia: findings from the STRATA study

**DOI:** 10.1038/s41537-022-00265-5

**Published:** 2022-07-12

**Authors:** Kira Griffiths, Alice Egerton, Edward Millgate, Adriana Anton, Gareth J. Barker, Bill Deakin, Richard Drake, Emma Eliasson, Catherine J. Gregory, Oliver D. Howes, Eugenia Kravariti, Stephen M. Lawrie, Shôn Lewis, David J. Lythgoe, Anna Murphy, Philip McGuire, Scott Semple, Charlotte Stockton-Powdrell, James T. R. Walters, Stephen R. Williams, James H. MacCabe

**Affiliations:** 1 Department of Psychosis Studies, Institute of Psychiatry, Psychology & Neuroscience, King’s College London, London, SE5 8AF UK; 2 NIHR Biomedical Research Centre at South London and Maudsley NHS Foundation Trust, London, UK; 3 Division of Neuroscience and Experimental Psychology, School of Biological Sciences, Faculty of Biology, Medicine and Health, University of Manchester, Manchester, M13 9PL UK; 4 Academic Radiology, Department of Infection, Immunity and Cardiovascular Disease, Medical School, Faculty of Medicine, Dentistry and Health, University of Sheffield, Sheffield, S10 2JF UK; 5 Department of Neuroimaging, Institute of Psychiatry, Psychology & Neuroscience, King’s College London, London, SE5 8AF UK; 6 Faculty of Biology, Medicine and Health, University of Manchester, Manchester, M13 9PL UK; 7 Greater Manchester Mental Health NHS Foundation Trust, Manchester, M25 3BL UK; 8 Division of Psychiatry, University of Edinburgh, Edinburgh, EH10 5HF UK; 9 Psychiatric Imaging Group MRC London Institute of Medical Sciences, Hammersmith Hospital, London, W12 0NN UK; 10 BHF Centre for Cardiovascular Science, University of Edinburgh, Edinburgh, EH16 4TJ UK; 11 Division of Informatics, Imaging and Data Sciences, School of Health Sciences, Faculty of Biology, Medicine and Health, University of Manchester, Manchester, M13 9PL UK; 12 MRC Centre for Neuropsychiatric Genetics and Genomics, Division of Psychological Medicine and Clinical Neurosciences, School of Medicine, Cardiff University, Cardiff, CF24 4HQ UK; 13 Division of Informatics, Imaging and Data Sciences, University of Manchester, Manchester, M13 9PL UK

**Keywords:** Biomarkers, Molecular neuroscience

## Abstract

Impaired cognition is associated with lower quality of life and poor outcomes in schizophrenia. Brain glutamate may contribute to both clinical outcomes and cognition, but these relationships are not well-understood. We studied a multicentre cohort of 85 participants with non-affective psychosis using proton magnetic resonance spectroscopy. Glutamate neurometabolites were measured in the anterior cingulate cortex (ACC). Cognition was assessed using the Brief Assessment for Cognition in Schizophrenia (BACS). Patients were categorised as antipsychotic responders or non-responders based on treatment history and current symptom severity. Inverted U-shaped associations between glutamate or Glx (glutamate + glutamine) with BACS subscale and total scores were examined with regression analyses. We then tested for an interaction effect of the antipsychotic response group on the relationship between glutamate and cognition. ACC glutamate and Glx had a positive linear association with verbal memory after adjusting for age, sex and chlorpromazine equivalent dose (glutamate, *β* = 3.73, 95% CI = 1.26–6.20, *P* = 0.004; Glx, *β* = 3.38, 95% CI = 0.84–5.91, *P* = 0.01). This association did not differ between good and poor antipsychotic response groups. ACC glutamate was also positively associated with total BACS score (*β* = 3.12, 95% CI = 0.01–6.23, *P* = 0.046), but this was not significant after controlling for antipsychotic dose. Lower glutamatergic metabolites in the ACC were associated with worse verbal memory, and this relationship was independent of antipsychotic response. Further research on relationships between glutamate and cognition in antipsychotic responsive and non-responsive illness could aid the stratification of patient groups for targeted treatment interventions.

## Introduction

Impaired cognition in psychotic disorders contributes to poor social and functional outcomes^1–3^. Cognitive deficits are observed in relatives of schizophrenia patients, clinical high-risk groups, and at the onset of psychosis^4–6^. Cognitive dysfunction is therefore an important target for research as it may precede potential confounds of prolonged antipsychotic treatment and illness chronicity. Antipsychotics primarily act as dopamine D2 receptor antagonists and have minimal impact on alleviating cognitive dysfunction^7–10^. This suggests that cognitive impairment in psychosis involves mechanisms other than dopamine. Investigating mechanisms of impaired cognitive function can advance our understanding of the aetiology of illness and the development of targeted treatments.

Converging lines of evidence implicate altered glutamatergic function in the aetiology of schizophrenia, particularly for negative and cognitive symptom domain^11–13^. A primary model of glutamate dysregulation centres on NMDA receptor hypofunction, which leads to excessive signalling of glutamatergic pyramidal neurons across the cortex and elevated glutamate release^14^. Administration of NMDA receptor antagonists increases cortical glutamate^15–17^, induces schizophrenia-like cognitive deficits in animal models and healthy human subjects^18–22^ and exacerbates cognitive impairment in schizophrenia^23,24^. Further, animal models provide some indication that cognitive impairments induced by NMDA antagonists may be partially reversed by moderate doses of glutamate modulating compounds^25^. The clinical efficacy of several compounds enhancing NMDA receptor signalling has been trialled, with meta-analysis demonstrating a small effect size (ES) for the reduction of PANSS-cognitive symptoms (ES = 0.28)^26^. One other meta-analysis found no evidence of improved cognitive function from antipsychotic treatment augmented with glutamatergic modulators^27^, which highlights the difficulties in translating findings from preclinical studies into effective glutamate drug therapies.

The relationship between glutamate and cognition in schizophrenia is not yet understood^28,29^. The anterior cingulate cortex (ACC) is involved in cognition^30^ and demonstrates abnormal activity during cognitive task performance in schizophrenia^31^. Six studies have examined the relationship between ACC glutamate neurometabolites and cognition in medicated patients^32–37^. One study reported a positive association between Glx and a composite measure of neurocognitive scores^37^. Three other studies assessed cognition across multiple domains using the Repeatable Battery for Neuropsychological Status (RBANS) and reported no association between glutamate or Glx (glutamate plus glutamine) concentrations and cognitive performance^33,34,36^. Another study found no evidence of an association between glutamate and performance on working memory and processing speed^35^. The sixth study found a positive association between Glx and cognitive flexibility, measured using the Wisconsin Card Sorting Task^32^. A positive association between dorsal ACC Glx and measures of working memory and attention was also reported in a large unmedicated patient sample^38^. Within the medial prefrontal cortex more broadly, no association between Glx and working memory was found in a sample of medicated and unmedicated patients^39^, whilst another study reported a negative association between the ratio of glutamine to glutamate and measures of cognitive flexibility, verbal working memory and attention^40^. Notably, the majority of these investigations included relatively small sample sizes and did not account for potential confounding effects of age, sex and antipsychotic dose on brain glutamate concentrations^41,42^.

Brain glutamate concentrations may vary across patients and there is some evidence that elevations in ACC glutamate neurometabolites are associated with a higher illness severity, worse clinical course, poorer functioning and treatment resistance^36,42–49^. Neuroimaging studies provide some evidence that when cortical glutamatergic metabolite concentrations are higher within schizophrenia groups compared to healthy controls the direction of associations between glutamate and cognition are negative^29,50^. Conversely, in studies where cortical glutamatergic metabolite concentrations are comparable or lower in schizophrenia groups than in healthy controls, associations between glutamate and cognition are positive^32,37,51–54^. One explanation for these different findings may be that deficient or excess cortical glutamate beyond some optimal range leads to cognitive impairments in schizophrenia. Taken together, these findings could potentially suggest an inverted ‘U’ shaped relationship between glutamate and cognition in schizophrenia.

One study has examined the relationship between glutamate and cognition in treatment-resistant and treatment-responsive illness, which found no relationship between dorsal ACC glutamate and cognition across the whole sample or within the patient subgroups^36^. However, the study used linear correlational analysis, which meant potential non-linear relationships between glutamate and cognition were not assessed and possible confounds were not controlled for. Further, although ACC Glx was higher in the treatment-resistant group compared to healthy controls, the difference in Glx between treatment and treatment-responsive groups did not differ significantly, which may explain why the relationships between Glx and cognition also did not differ between groups^36^.

This study investigated the relationship between ACC glutamatergic metabolites and cognitive data in the STRATA-1 patient cohort, who were recruited according to their response to antipsychotic treatment. Within this cohort we previously reported higher glutamate levels in the ACC of antipsychotic non-responders compared to responders when controlling for age and sex^45^, but no group differences in cognition^55^. Based on the evidence above, our primary hypothesis was that there would be an inverted U-shaped association between ACC glutamate and cognition across the whole sample. Second, we hypothesised an interaction effect of antipsychotic response group on the relationship between glutamate and cognition, such that the association would be positive in antipsychotic responders and negative in antipsychotic non-responders.

## Results

Demographic and clinical characteristics of the cohort are reported in Table 1. In total, 85 participants had both ^1^H-MRS and cognitive data. One participant did not complete the task assessing motor processing speed (Token Motor Task). All other participants completed each task. The mean number of days between the collection of both measures was 4 (SD = 8.00, range = 0–40 days). An example MR spectrum is presented in Supplementary Fig. 1.

**Table 1:** Sample characteristics of the combined ^1^H-MRS and cognition cohort.

	*n* = 85
Sex male/female	71/14
Age (years)	29.47 ± 8.29
Age of onset (years)	24.44 ± 6.47
Duration of illness (years)	4.83 ± 6.30
Diagnosis psychosis unspecified/schizophrenia/delusional disorder	21/63/1
Ethnicity White/Black/Mixed White Black/Asian/Other	44/22/5/7/7
Benzodiazepine yes/no	Aug-77
Antidepressant yes/no	13/72
Current smoking no/less than daily/daily	35/5/45
Current cannabis yes/no	Oct-75
CPZE dose (mg/day)	456.54 ± 298.39
Current antipsychotic	
Amisulpride	6
Aripiprazole	18
Clopixol	3
Paliperidol	4
Haloperidol	1
Flupenthixol	2
Olanzapine	20
Quetiapine	11
Risperidone	13
Combination	7
Symptom severity	
PANSS total	68.92 ± 18.6
PANSS positive	16.88 ± 6.16
PANSS negative	17.24 ± 5.61
PANSS general	34.80 ± 9.15
Cognition	
Verbal memory	39.41 ± 11.10
Verbal fluency	31.53 ± 9.10
Working memory	18.31 ± 4.33
Attention & information processing speed	47.25 ± 11.17
Motor speed*	67.82 ± 14.67
Executive function	16.42 ± 4.07
BACS-t*	31.41 ± 13.04
BACS-z*	−1.84 ± 1.32
Glutamate neurometabolites	
ACC Glu_corr_ (total, *n* = 85)	−0.01 ± 0.97
CU (*n* = 8)	13.07 ± 1.97
UoE (*n* = 12)	14.17 ± 3.37
UoM (*n* = 31)	15.22 ± 4.14
KCL (*n* = 34)	17.52 ± 3.85
CRLB	5.82 (0.88)
ACC Glx_corr_ (total, *n* = 85)	−0.01 ± 0.98
CU (*n* = 8)	17.44 ± 3.21
UoE (*n* = 12)	20.76 ± 4.61
UoM (*n* = 31)	21.70 ± 5.87)
KCL (*n* = 34)	23.12 ± 4.46)
CRLB	6.18 (1.36)

*Age* age at study enrolment, *CPZE* chlorpromazine equivalent dose, *ACC* anterior cingulate cortex, *CU* Cardiff University, *UoE* Edinburgh University, *UoM* University of Manchester, *KCL* King’s College London, *CRLB* Cramér–Rao lower bounds.

Data are expressed as mean ± unless otherwise stated. Site-specific glutamatergic metabolite concentrations are expressed as absolute values corrected for voxel tissue content. Total glutamatergic metabolite concentrations are corrected for voxel tissue content and expressed as z-scores.

*n = 84 because one participant did not complete the Token Motor Task and therefore also had no composite (t or z) scores.

### The relationship between cognition and clinical and demographic variables

Supplementary Table 1 reports the relationship between cognition and clinical and demographic characteristics of the sample. Sex, age of onset, CPZE dose, current cannabis use, current smoking, current benzodiazepine and current SSRI use were not associated with BACS composite t and z or subdomain scores. Age was negatively correlated with motor speed and attention and information processing speed. BACS composite t and z-scores, verbal memory, verbal fluency, working memory and attention and information processing speed were negatively correlated with PANSS negative subscale scores (Supplementary Table 2). As reported in our recent publication in the same cohort^55^, there was no difference in cognitive performance between groups prescribed antipsychotics with no or low versus high anticholinergic effects (see ref. ^55^ and Supplementary Materials).

### Cognition and glutamatergic metabolites

**Fig. 1:**
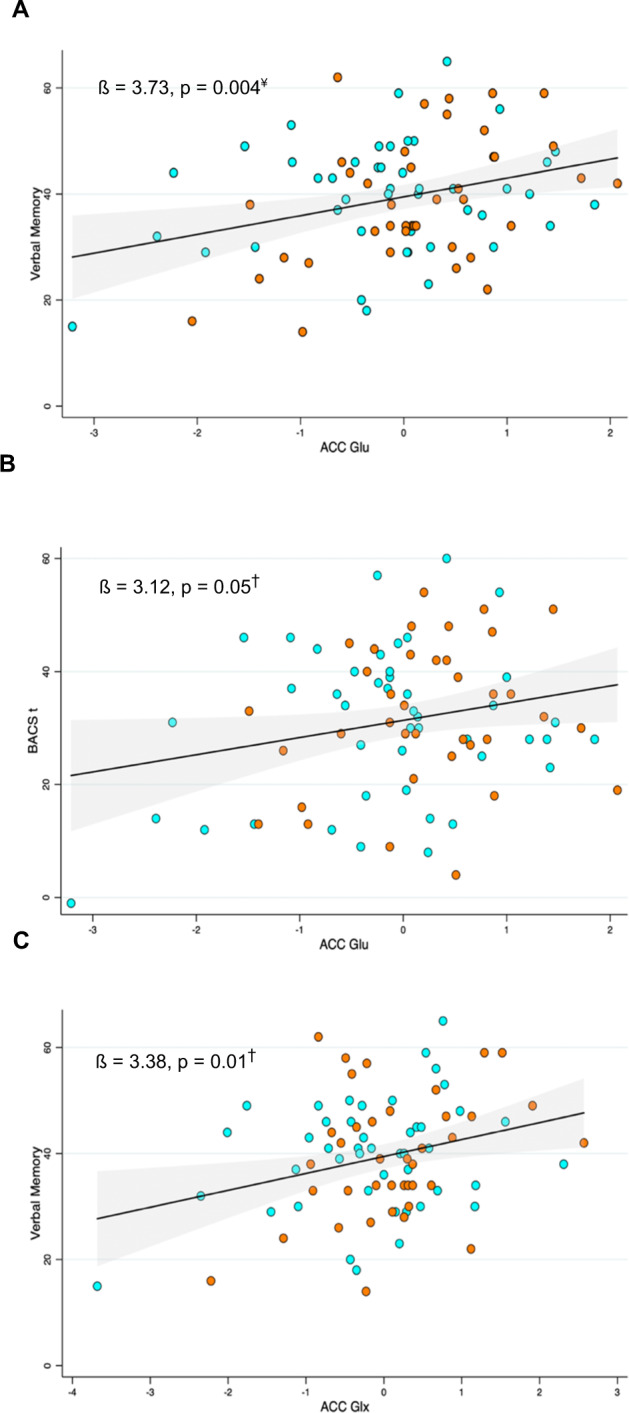
Scatter plots of ACC glutamatergic metabolites and cognition (BACS-t and verbal memory). ^¥^Linear regression model adjusted for age and sex; ^†^linear regression model adjusted for age, sex and CPZE.

Tables 2 and 3 display results from all multivariable regression models. The direction of relationship between ACC Glu_corr_ and Glx_corr_ with cognition was positive across all cognitive domains (Supplementary Figs. 2 and 3). ACC Glu_corr_ and Glx_corr_ were positively associated with verbal memory after adjusting for age and sex. Both associations remained significant after adjusting for CPZE (Glu_corr_, *β* = 3.73, 95% CI = 1.26–6.20, *P* = 0.004; Glx_corr_, *β* = 3.38, 95% CI = 0.84–5.91, *P* = 0.01). Visual inspection of the data indicated a linear relationship between glutamate neurometabolites and verbal memory (Fig. 1A, C); non-linear associations for both Glu_corr_ and Glx_corr_ with verbal memory were not significant (Glu_corr_^2^: *β* = −0.87, 95% CI = −2.42, 0.68, *P* = 0.27; Glx_corr_^2^: *β* = −0.65, 95% CI = −1.91, 0.61, *P* = 0.31) and the LR test suggested a linear model was a better fit to data (Glu_corr_: LR X^2^ (1) = 1.33, *P* = 0.25; Glx_corr_: LR X^2^ (1) = 1.12, 0.29). There was no evidence of an inverted U-shaped relationship between glutamate (*t* = 0.10, *P* = 0.46) or Glx (*t* = 0.07, *P* = 0.47) and verbal memory. Subsequent analyses including an *antipsychotic response group × metabolite* interaction term found no significant effects, such that the relationship between verbal memory and Glu_corr_ or Glx_corr_ did not differ as a function of antipsychotic response group (Glu_corr_ × group: *β* = 3.43, 95% CI = −1.51, 8.36, *P* = 0.17; Glx_corr_ × group: *β* = 1.12, 95% CI = −3.92, 6.16, *P* = 0.66).

**Table 2:** Linear regression models with ACC glutamate as the independent variable of interest, predicting cognitive performance.

	Unadjusted			Model 1			Model 2		
	*β*	95% CI	*P*	*β*	95% CI	*P*	*β*	95% CI	*P*
Dependent variable: cognitive outcome									
Verbal memory	3.54	1.18, 5.90	0.004*	3.74	1.29, 6.20	0.002*	3.73	1.26, 6.20	0.004*
Verbal fluency	1.65	−0.35, 3.64	0.11	2.01	−0.07, 4.09	0.06	1.96	−0.12, 4.04	0.07
Working memory	1.00	0.06, 1.94	0.04*	0.70	−0.28, 1.67	0.16	0.70	−0.29, 1.68	0.16
Attention & information processing speed	1.52	−0.95, 3.99	0.23	0.98	−1.59, 3.55	0.45	0.91	−1.66, 3.47	0.48
Motor speed	2.80	−0.43, 6.03	0.09	3.14	−0.86, 6.36	0.06	3.09	−0.15, 6.34	0.06
Executive function	0.75	−0.15, 1.64	0.10	0.59	−0.35, 1.53	0.21	0.61	−0.34, 1.56	0.21
BACS-t	3.05	0.12, 5.97	0.04*	3.12	0.01, 6.23	0.05*	3.05	−0.08, 6.17	0.06
BACS-z	0.28	−0.01, 0.58	0.06	0.31	−0.01, 0.62	0.06	0.30	−0.02, 0.61	0.06

*P ≤ 0.05.

Model 1 adjusts for age and sex. Model 2 adjusts for age, sex and CPZE dose.

**Table 3:** Linear regression models with ACC Glx as the independent variable of interest, predicting cognitive performance.

	Unadjusted			Model 1			Model 2		
	*β*	95% CI	*P*	*β*	95% CI	*P*	*β*	95% CI	*P*
Dependent variable: cognitive outcome									
Verbal memory	3.19	0.83, 5.56	0.009*	3.40	0.88, 5.91	0.009*	3.38	0.84, 5.91	0.01*
Verbal fluency	0.58	−1.44, 2.59	0.57	0.84	−1.30, 2.99	0.44	0.77	−1.38, 2.92	0.48
Working memory	1.04	0.10, 1.97	0.03*	0.69	−0.30, 1.68	0.17	0.69	−0.30, 1.69	0.17
Attention & information processing speed	2.16	−0.27, 4.59	0.08	1.58	−1.01, 4.17	0.23	1.48	−1.11, 4.07	0.26
Motor speed	2.80	−0.41, 6.03	0.09	3.10	−0.19, 6.39	0.06	3.05	−0.27, 6.37	0.07
Executive function	1.03	0.15, 1.90	0.02*	0.90	−0.04, 1.84	0.06	0.92	−0.03, 1.87	0.06
BACS-t	2.86	−0.07, 5.80	0.06	2.98	−0.20, 6.16	0.07	2.88	−0.33, 6.08	0.08
BACS-z	0.26	−0.03, 0.56	0.08	0.29	−0.03, 0.61	0.08	0.28	−0.05, 0.60	0.09

*P < 0.05.

Model 1 adjusts for age and sex. Model 2 adjusts for age, sex and CPZE dose.

There was a significant association between ACC Glu_corr_ and BACS-t scores when controlling for age and sex (*β* = 3.12, 95% CI = 0.01, 6.23, *P* = 0.049). Visual inspection of the data indicated a linear relationship between glutamate neurometabolites and BACS-t (Fig. 1). Consistent with visual inspection, the non-linear association between Glu_corr_ and BACS-t was not significant (Glu_corr_^2^: *β* = −2.25, 95% CI = −5.07, 0.57, *P* = 0.17), the LR test suggested a linear model was a better fit to the data (LR X^2^ (1) = 4.77, *P* = 0.09) and the utest confirmed no evidence of an inverted U-shaped relationship between Glu_corr_ and BACS-t (*t* = 1.31, *P* = 0.10). When the linear model was further adjusted for CPZE, the association between BACS-t and Glu_corr_ became non-significant. There was no significant relationship between BACS-t and ACC Glx_corr_ when controlling for age, sex and CPZE. No significant *antipsychotic response group × metabolite* interaction effects were found for BACS-t scores and performance on all other cognitive domains (Supplementary Table 3 and Supplementary Figs. 4 and 5).

One participant had low values on both BACS-t and ACC Glu_corr_ (Fig. 1B). Both raw data entry and the BACS-t composite score calculation were checked for accuracy. We did not exclude this participant from analyses because, relative to the overall STRATA-1 cognition cohort, the BACS-t score fell within the range of the sample^55^. ^1^H-MRS spectra were visually inspected, and the scan passed all our standard quality control procedures^45^.

## Discussion

This study investigated the relationship between glutamatergic metabolites and cognition in a sample of medicated participants with non-affective psychosis. We also explored whether there was a group difference in the relationship between glutamatergic metabolites and cognition between antipsychotic responders and non-responders. Our main finding was a positive linear association between verbal memory performance and both ACC Glu_corr_ and Glx_corr_ after adjusting for age, sex and CPZE dose. Interaction analyses found no effect of antipsychotic response on the relationship between Glu_corr_ or Glx_corr_ and verbal memory. ACC Glu_corr_ was also associated with BACS-t after controlling for age and sex, but further adjustments for CPZE dose rendered this association non-significant. We did not find evidence to support the hypothesised inverted U-shaped relationship between glutamate and cognition. Overall, our results indicate that higher levels of glutamate may be associated with better verbal memory performance in schizophrenia and that this relationship does not differ according to the degree of antipsychotic response.

The finding of a positive association between ACC glutamatergic metabolites and verbal memory performance is broadly consistent with some previous studies on unmedicated first episode psychosis and chronically medicated schizophrenia cohorts, which have reported positive associations between ACC Glx and measures of working memory, attention and executive function^32,37,38^. Conversely, four other studies on medicated cohorts have reported no association between ACC glutamatergic metabolites and performance on tasks measuring broad neurocognitive status, working memory and information processing speed^33–36^. Most of these investigations included relatively small samples and did not control for possible effects of age, sex and antipsychotic dose were not controlled for. Results from the largest medicated cohort published to date (*n* = 104 schizophrenia patients, 97 healthy controls)^56^ also found no significant association between performance across the MATRICS^57^ cognitive battery and Glx concentrations in supraventricular white matter regions, which included a portion of the ACC but was not limited to this brain region^56^. Overall, between-study differences in the cognitive tasks employed, method of adjusting glutamatergic metabolites (scaling to creatine or correcting for voxel tissue content), neural region of interest (including voxel positioning in the ACC) and the clinical characteristics of the patient samples make direct comparisons difficult.

We found that higher glutamate was associated with better verbal memory across the whole cohort. This finding appears to be at odds with data from animal and human studies which suggest that compounds that increase cortical glutamate levels disrupt cognitive function^19,21–25^. Further, this finding also conflicts with observations that schizophrenia groups displaying the highest levels of illness severity, including cognitive dysfunction, may also have higher brain glutamate than less symptomatic patients and healthy controls^18,36,46,47^. However, neuroimaging studies collectively suggest that the relationship between glutamate and cognition may vary depending on whether glutamate in schizophrenia is elevated or comparable to healthy controls^32,38,40,51–54,56,58^. Preclinical models and proof-of-concept clinical studies also give some indication that glutamate modulating compounds improve cognition at moderate doses, whereas low and high doses may have suboptimal or negative effects, respectively, on cognition^25,59–61^. These observations raise the possibility that insufficient or excessive glutamate outside of an optimal range may be associated with worse cognitive functioning. Within our sample the positive relationship between cognition and glutamate appeared linear, which would be consistent with findings in patient cohorts with glutamate levels comparable or lower to those in healthy controls^32,38,51–54^. Future research could specifically examine whether the relationship between glutamate and cognition differs in patients with glutamate levels above or below healthy control values.

The largest effect size was found for the association between glutamate and verbal memory. Impairments within verbal cognitive domains are robust across the illness course of schizophrenia^62,63^. Impaired verbal memory function is also evident in the clinical high-risk phase of psychosis and is more severe in high-risk subjects who later progress to psychosis^64,65^. A recent meta-analysis found that verbal memory impairments are more apparent in treatment-resistant compared to treatment-responsive schizophrenia^66^, and prospective investigation suggests this group difference is detectable from the first episode of psychosis^67^. However, cognitive performance did not differ between the good and poor antipsychotic response groups in our cohort^55^. We found no evidence that the relationship between glutamate and verbal memory differed between good and poor antipsychotic responders. Whilst both worse verbal cognition and higher ACC glutamate may be associated with treatment resistance^66–68^, the lack of interaction suggests that there is no qualitative difference in the relationship between glutamate and cognition between antipsychotic response groups, at least within the ranges of antipsychotic response, glutamate, and cognitive function measured in the current study. Alternatively, it is possible that the lack of group difference in the relationship between glutamate and cognition may be due to the relative similarity in cognitive function between good and poor antipsychotic responders in our sample^55^. One other study found no association between ACC Glx and cognition within treatment-resistant and -responsive groups despite the treatment-resistant sample having worse cognitive function^36^. However, patient groups did not differ in Glx concentrations and the interaction between group and glutamatergic metabolite concentrations on cognition was not directly investigated. Whether the marked cognitive impairments observed in treatment-resistant schizophrenia^66^ represent a distinct or exaggerated pathophysiological mechanism compared to those displaying good treatment response warrants further investigation.

### Strengths and limitations

We present data from a multicentre investigation, which provides a representative sample of medicated patients across the UK and a larger sample size than most previous studies. We used the BACS to assess cognitive function within several domains directly relevant to schizophrenia^69,70^, which is quicker to administer than other broad neuropsychological batteries (~30 min versus a couple of hours) whilst remaining as sensitive in detecting cognitive impairments^69^. ^1^H-MRS acquisition sequences were harmonised across research sites, and we were able to control for site effects present in MRS data by standardising metabolite values. Further, in vitro phantom data and a healthy control pilot scan confirmed good data quality across research sites (see ref. ^45^ and Supplementary Discussion).

While several statistical tests were run, we did not correct for multiple comparisons given preclinical and human imaging evidence for associations between glutamate and task performance measured across several independent cognitive domains^18–24,29^. In terms of study limitations, not all brain scans and cognitive assessments were performed on the same day, which may have affected the relationship we observed between glutamate and cognition. Further, our study did not include the measurement of glutamatergic metabolites in other brain regions relevant for cognition, such as the dorsolateral prefrontal cortex and the hippocampus^29^. Another caveat is that CPZE dose does not account for variations in medication adherence, although we only recruited participants demonstrating at least a moderate level of adherence by applying the CRS scale. Future research would benefit from the collection of antipsychotic plasma levels to confirm treatment adherence and exclude cases of pseudo-treatment resistance. It is also important to note that our patient sample had an average illness duration of 5 years and therefore we cannot make inferences on the relationship between glutamate and cognition in initial stages of illness or the first episode of psychosis. This is important given evidence that the relationship between glutamate and cognition may change after antipsychotic treatment^71^, although how this may also relate to antipsychotic response is still unstudied.

## Conclusion

This study found a positive association between glutamate and cognition in schizophrenia and this relationship did not differ between good and poor antipsychotic responders. Our findings support a role of ACC glutamate for cognition in schizophrenia.

## Methods

### Ethics

The study had NHS Research Ethics Committee approvals (15/LO/0038). All participants provided written informed consent.

### Participants

Participants were recruited across four sites: Cardiff University (CU), University of Edinburgh (UoE), University of Manchester (UoM) and King’s College London (KCL). Inclusion criteria were being aged between 18 and 65, meeting Diagnostic and Statistical Manual of Mental Disorders (DSM-5) criteria for schizophrenia or schizophreniform disorder and the ability to understand and consent to study procedures, including a sufficient level of English. Exclusion criteria were poor medication adherence (defined as a score of <3 on the Compliance Rating Scale (CRS))^72^, current pregnancy, previous severe head injury involving loss of consciousness for >5 min, currently meeting the international Classification of Diseases (ICD) criteria for harmful substance misuse or psychotic disorder secondary to substance misuse and any Magnetic Resonance imaging (MRI) contraindications, such as implanted electronic devices or metallic objects. Participants were also excluded if they had received treatment with clozapine in the last 3 months prior to study screening because clozapine may affect brain glutamate concentrations^73^.

This patient group is a subsample of the STRATA-1 imaging cohort presented in a previous publication^45^. As well as a brain MRI scan with proton magnetic resonance spectroscopy (^1^H-MRS), participants included in the current study completed a battery of cognitive assessments^55^. Scans and cognitive assessments were performed on the same day where possible. For each antipsychotic dose, chlorpromazine equivalent (CPZE) doses were calculated according to methods outlined by Davis and Chen^74^, except for amisulpride which used defined daily dose (https://www.whocc.no/atc_ddd_index/).

### Defining good and poor antipsychotic response

Participants displaying good and poor antipsychotic responses were recruited based on a priori criteria for antipsychotic treatment response. Recruitment aimed for a 1:1 ratio of antipsychotic responders and non-responders. Treatment history and current symptom severity were assessed through structured interviews and review of medical records. The antipsychotic response was defined as (1) having treatment with only one antipsychotic since illness onset, or treatment changes that were due to adverse effects rather than non-response; (2) clinical Global Impression-Schizophrenia Scale (CGI-SCH) severity score <4; (3) positive and Negative Syndrome Scale (PANSS) total score <60. Antipsychotic non-response was defined as (1) documented treatment with at least two antipsychotics for >4 weeks each, at doses above the minimum therapeutic doses as defined by the British National Formulary; (2) a CGI-SCH score >3; (3) PANSS total score of at least 70.

### ^1^H-MRS and quality control procedures

Metabolite concentrations were measured using ^1^H-MRS at 3 Tesla according to the protocol described by Egerton and colleagues^45^. Spectra were analysed in LCModel version 6.3.1 L using a standard LCModel basis set. Extracted metabolite estimates were water-referenced and corrected for voxel tissue content (denoted by Glu_corr_ and Glx_corr_)^45^. We chose not to scale metabolite values to creatine given evidence that these metabolite values may vary in schizophrenia cohorts^42^. The spectral linewidths and signal-to-noise ratio were reviewed as part of quality control procedures, and spectra were excluded if the linewidth was 2 standard deviations above or the signal-to-noise ratio was 2 standard deviations below the overall mean for the voxel across all participants at all sites. Individual metabolite concentrations were excluded if their Cramér–Rao lower bounds (CRLB) value was 20% or higher. CRLBs for glutamate and Glx did not differ between good and poor antipsychotic response groups^45^, confirming that CRLB filtering did not result in different levels of exclusion of metabolite estimates between the groups^75^. All metabolite concentrations were converted to z-scores to account for site effects in metabolite concentration estimates, which were present due to differences in the MRI scanners used across sites (see ref. ^45^ and Supplementary Discussion for a detailed discussion of scanner and site effects).

### Cognition

Cognition was assessed using the Brief Assessment for Cognition in Schizophrenia^69^ (BACS). Performance was evaluated across six cognitive domains: executive function, working memory, motor processing speed, verbal memory, verbal fluency and attention and information process speed. Overall cognitive function was assessed using composite BACS-t and z-scores, which were standardised against normative data and calculated according to equations provided by Keefe and colleagues^55,70^. A z-score of 0 represents average performance with reference to the healthy control population of the same age range and sex, while each point represents 1 standard deviation. Higher scores reflect better cognitive performance on each domain and for the composite measures.

### Statistical analysis

All analyses were performed using STATAv.15^76^. Multivariable linear regression models were used to examine the effect of Glu_corr_ and Glx_corr_ on BACS composite and subdomain scores. Both composite and subdomain scores were considered as primary outcomes of interest given evidence of potential relationships between glutamate and cognition on several cognitive tasks measuring distinct processes^29^. Models were adjusted for age and sex based on their relationship with brain glutamate concentration^41,45,77^, To explore any potential effects of antipsychotic medication on glutamate and cognitive function^42,78,79^, CPZE dose was also entered as a covariate. We then tested whether a linear or non-linear model provided a better fit to our data by expanding models to include a quadratic term (metabolite^2^). We used the likelihood-ratio (LR) test to compare the fit of linear and non-linear models as well as the STATA command utest^80,81^ to examine whether non-linear relationships between metabolites and cognition were inverted U-shape. To test whether the relationship between glutamatergic metabolites and cognition differed between responder and non-responder groups, we subsequently included the interaction term *antipsychotic response group × metabolite* to fully adjusted linear models. All tests for statistical significance were two-sided with an alpha of 0.05.

## Supplementary information

Supplementary Materials. Impaired verbal memory function is related to anterior cingulate glutamate levels in schizophrenia: findings from the STRATA study

## Data Availability

The data that support the findings of this study are available from the corresponding authors upon reasonable request. At the time of submission, the data governance frameworks are being put in place to make a fully anonymized version of the data available to the wider research community via TranSMART data-sharing platform: https://transmartfoundation.org/, which will be hosted at King’s College London. To apply for access to the data, please contact the chief investigator J.H.M. at james.maccabe@kcl.ac.uk.
