# Supplementary material for: Impaired verbal memory function is related to anterior cingulate glutamate levels in schizophrenia: findings from the STRATA study
Source: Schizophrenia (Heidelb). 2022 Jul 12;8(1):60. doi: 10.1038/s41537-022-00265-5 (PMC9279335; doi:10.1038/s41537-022-00265-5)

**Impaired verbal memory function is related to anterior cingulate glutamate levels in schizophrenia: findings from the STRATA study**

**SUPPLEMENTARY MATERIALS**

**Figure S1. An example 1H-MRS spectrum in the Anterior Cingulate Cortex**

**
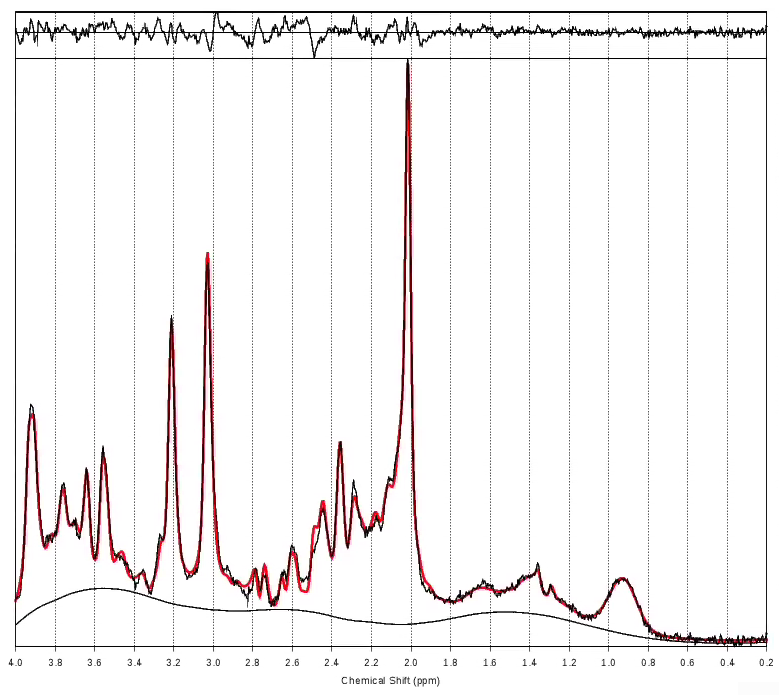
**

**Table S1. The relationship between cognition and clinical and demographic variables**

|  | Age | Sex | Age Onset | CPZE | Current Cannabis | Current Smoking | Current BDZ | Current ADP |
| --- | --- | --- | --- | --- | --- | --- | --- | --- |
| Verbal memory | -0.17 | -0.77 | -0.16 | -0.06 | -0.76 | -2.62 | -1.23 | -0.94 |
| Verbal fluency | -0.01 | -1.41 | -0.05 | -0.15 | 0.14 | -1.08 | 0.66 | 0.94 |
| Working memory | -0.20 | 1.66 | -0.09 | -0.02 | 1.01 | 0.75 | 0.38 | -0.90 |
| Attention & information processing speed | -0.24 | 0.46 | -0.12 | -0.15 | 0.02 | -0.77 | 0.36 | 0.49 |
| Motor speed | -0.30* | -1.97 | -0.14 | -0.09 | 0.21 | 0.46 | 0.79 | 0.33 |
| Executive function | -0.06 | 1.28 | -0.20 | 0.04 | 0.57 | -1.28 | -0.61 | 0.80 |
| BACS-t | -0.03 | 0.21 | -0.19 | -0.11 | -0.03 | -1.48 | 0.06 | -0.69 |
| BACS-z | -0.02 | -0.12 | -0.12 | -0.13 | -0.07 | -1.71 | 0.10 | -1.09 |

Statistics reported are Pearson’s r for continuous variables and ttest for categorical variables. CPZE = chlorpromazine equivalent dose; BDZ = benzodiazepine; ADP = antidepressant. For each demographic or clinical variable, p-values were adjusted for multiple comparisons.

* = p < 0.006

**Table S2. The relationship between cognition and symptom severity**

|  | PANSS-T | PANSS-P | PANSS-N | PANSS-G |
| --- | --- | --- | --- | --- |
| Verbal memory | -0.14 | -0.03 | -0.33** | -0.06 |
| Verbal fluency | -0.06 | 0.09 | -0.40*** | 0.07 |
| Working memory | -0.06 | 0.04 | -0.23* | 0.00 |
| Attention & information processing speed | -0.13 | 0.02 | -0.26* | -0.11 |
| Motor speed | 0.02 | 0.12 | -0.18 | 0.06 |
| Executive function | 0.00 | 0.11 | -0.19 | 0.04 |
| BACS-t | -0.05 | 0.12 | -0.32** | 0.02 |
| BACS-z | -0.02 | 0.14 | -0.32** | 0.05 |

* = p < 0.05, ** = p < 0.01, *** = p > 0.001

**Figure S2. Anterior Cingulate Cortex glutamate and cognition (BACS-z, verbal fluency, working memory, attention & information processing speed, motor speed, executive function)**


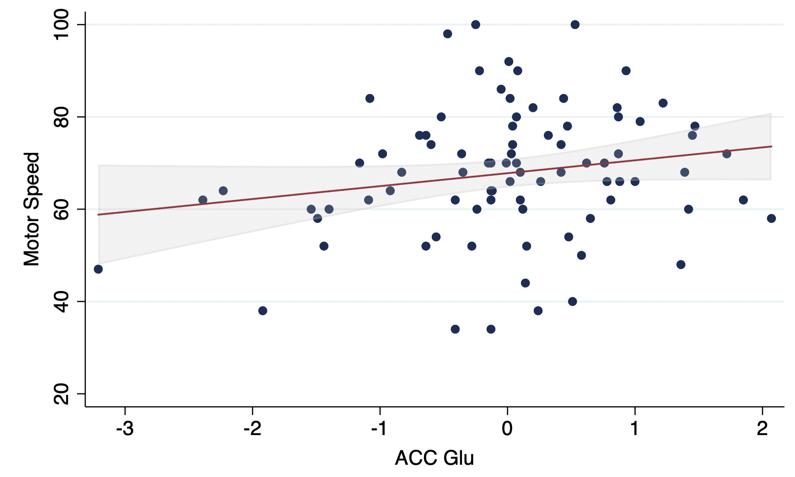

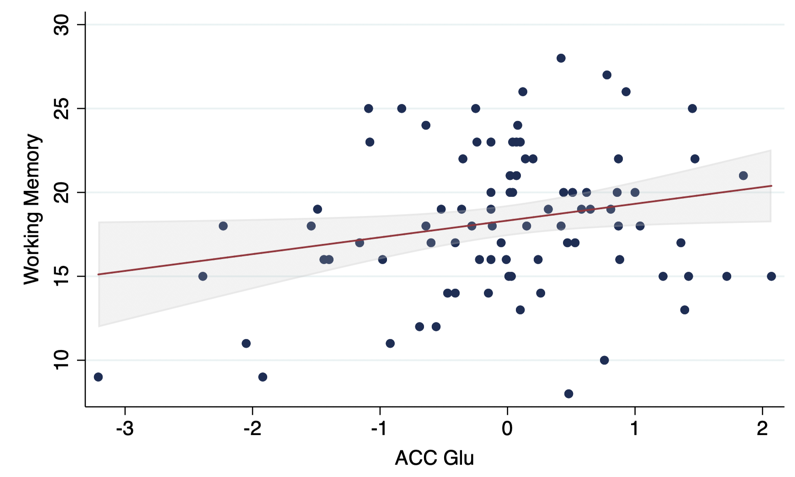

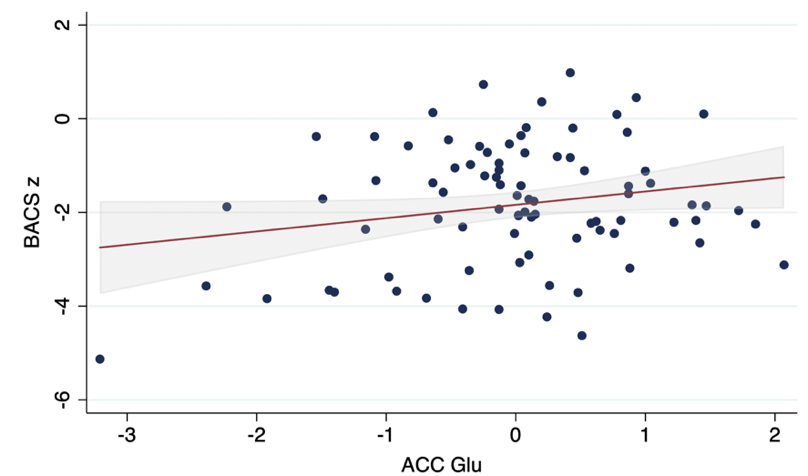


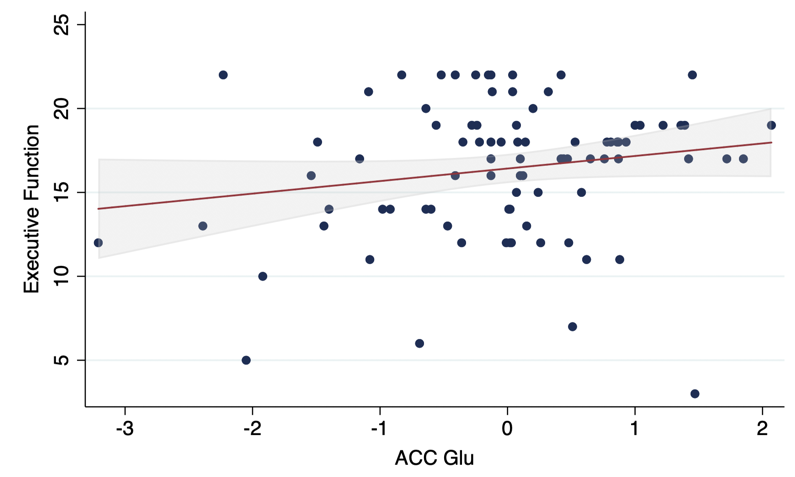

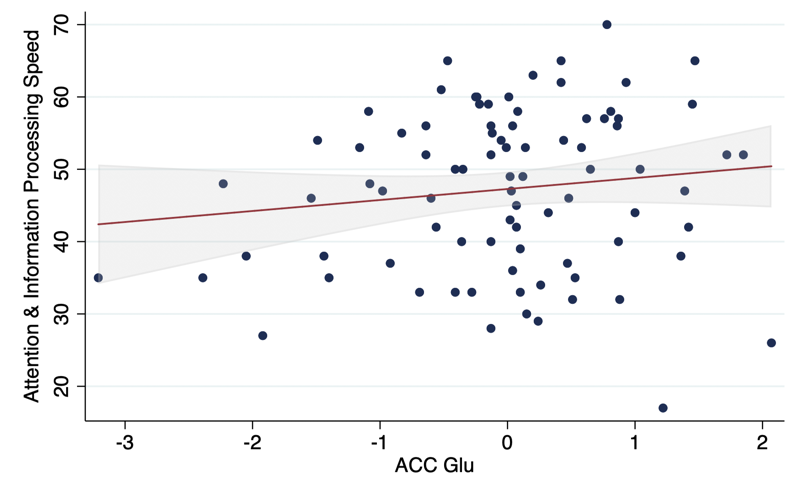

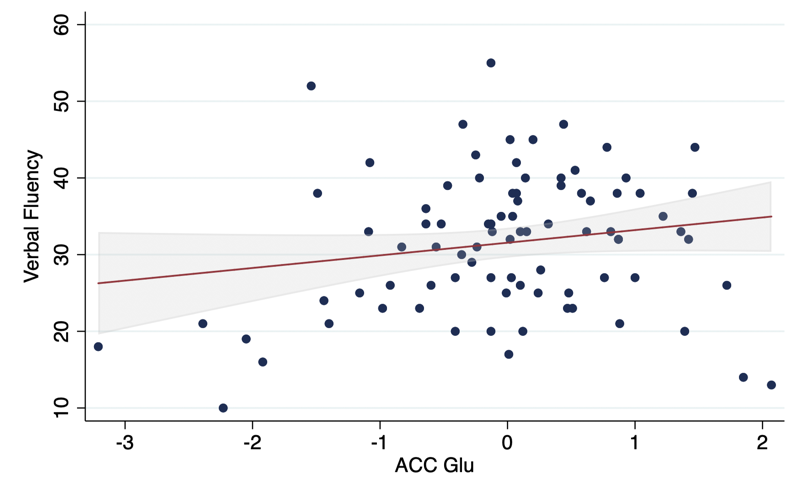


**Figure S3. Anterior Cingulate Cortex Glx and cognition (BACS-z, BACS-t, verbal fluency, working memory, attention & information processing speed, motor speed, executive function)**


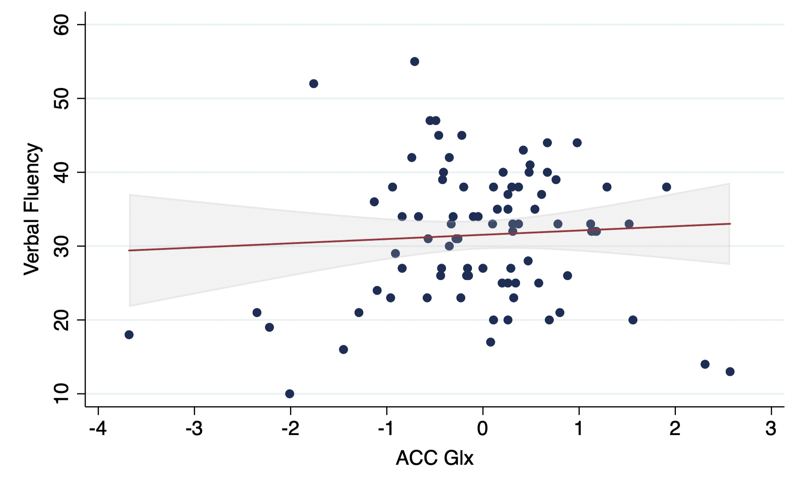

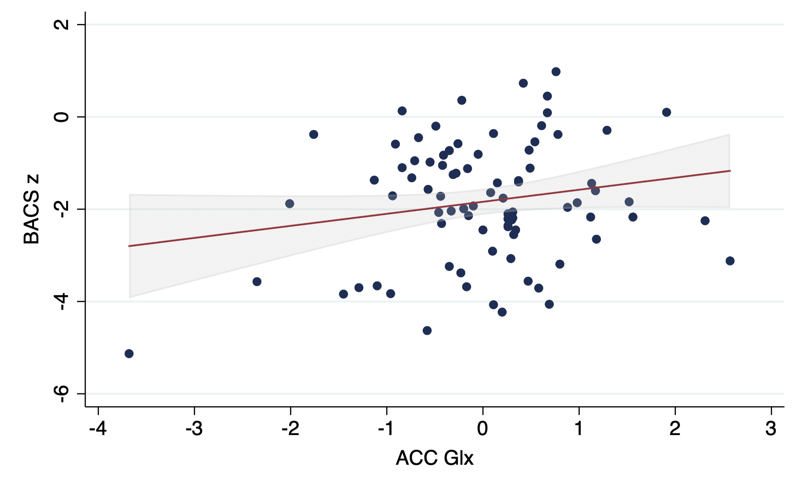

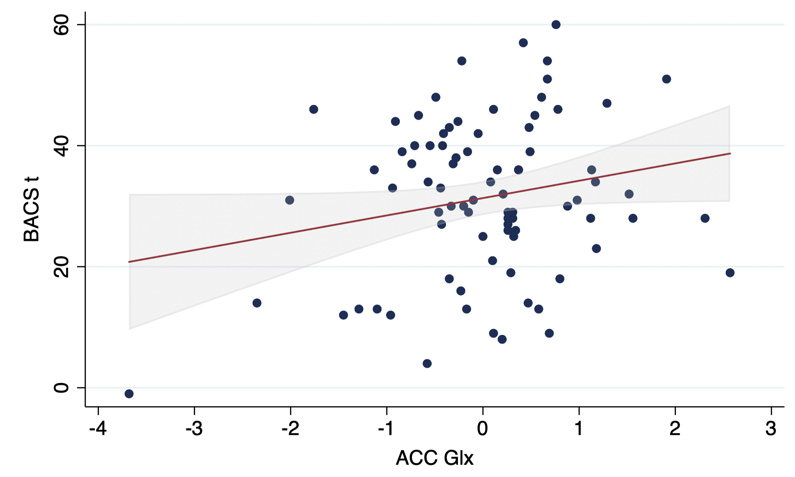


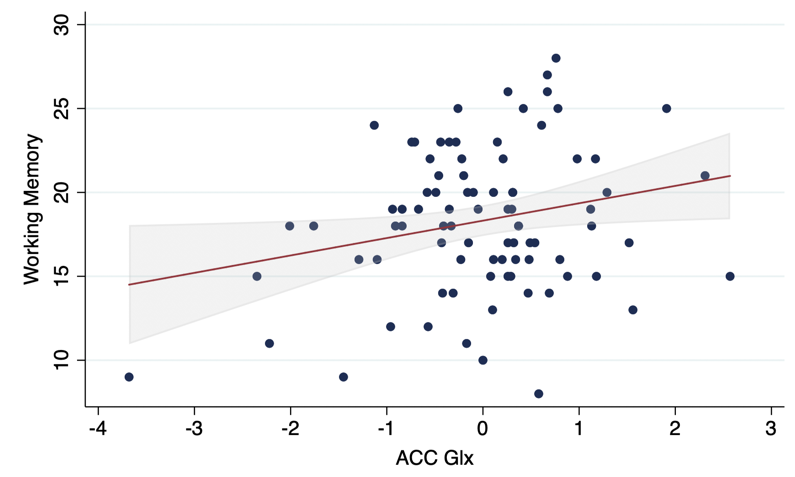

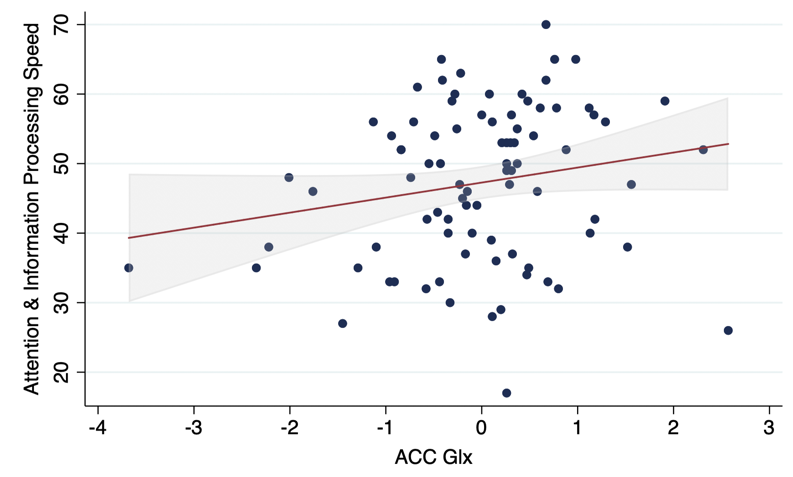

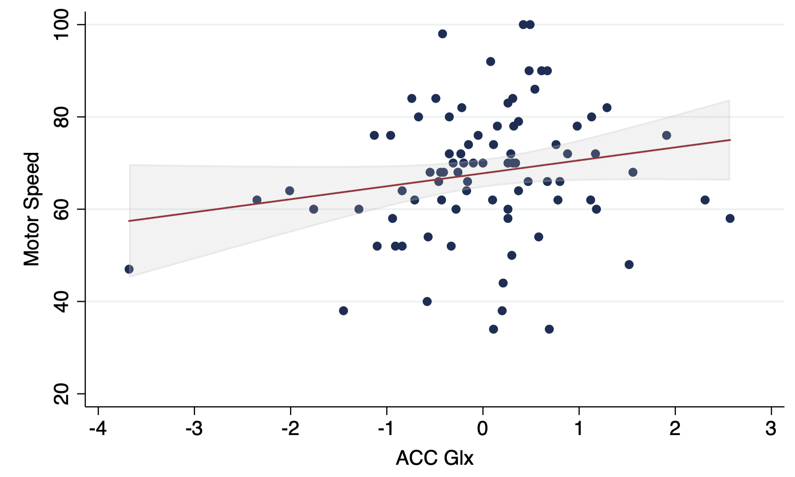


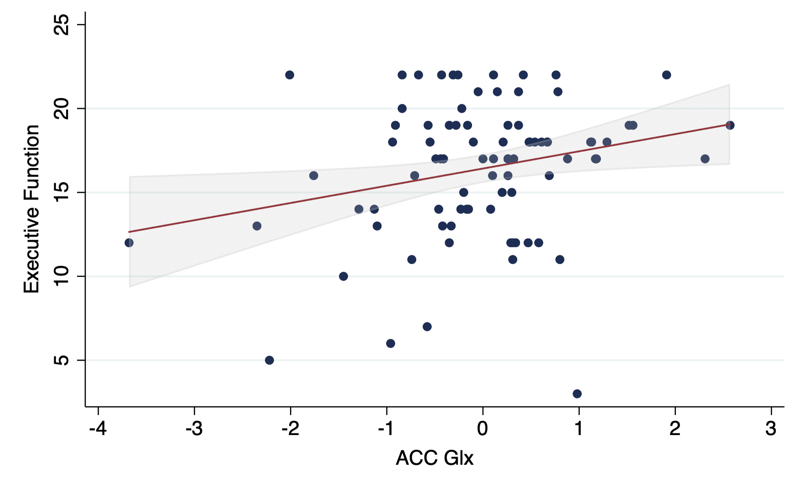


**Table S3. Results from linear regression analyses including group*metabolite interaction term. Glutamate or Glx are the independent variables predicting cognitive performance, adjusting for age, sex and CPZE**

|  | **ACC Glu** | | | **ACC Glx** | | |
| --- | --- | --- | --- | --- | --- | --- |
|  | **β** | **95% CI** | **p** | **β** | **95% CI** | **p** |
| **Verbal memory regression** | | | | | | |
| Metabolite | 2.54 | -0.67, 5.76 | 0.12 | 3.09 | -0.23, 6.42 | 0.07 |
| Group | -2.01 | -6.72, 2.70 | 0.40 | 1.12 | -6.59, 3.04 | 0.47 |
| Metabolite x Group | 3.43 | -1.51, 8.36 | 0.17 | 1.12 | -3.92, 6.16 | 0.66 |
| **Verbal fluency regression** | | | | | | |
| Metabolite | 2.18 | -0.57, 4.92 | 0.12 | 1.36 | -1.46, 4.18 | 0.34 |
| Group | 0.22 | -3.81, 4.26 | 0.91 | 0.67 | -3.41, 4.76 | 0.74 |
| Metabolite x Group | -0.59 | -4.81, 3.64 | 0.78 | -1.59 | -5.87, 2.69 | 0.46 |
| **Working memory regression** | | | | | | |
| Metabolite | 0.53 | -0.77, 1.82 | 0.42 | 0.77 | -0.54, 2.08 | 0.25 |
| Group | 0.36 | -1.54, 2.26 | 0.71 | 0.38 | -1.52, 2.28 | 0.70 |
| Metabolite x Group | 0.33 | -1.66, 2.32 | 0.74 | -0.28 | -2.27, 1.71 | 0.78 |
| **Attention & information processing speed regression** | | | | | | |
| Metabolite | 1.53 | -1.85, 4.91 | 0.37 | 2.43 | -0.96, 5.83 | 0.16 |
| Group | -0.20 | -5.16, 4.76 | 0.94 | -0.40 | -5.31, 4.52 | 0.87 |
| Metabolite x Group | -1.49 | -6.69, 3.71 | 0.57 | -2.22 | -7.37, 2.92 | 0.39 |
| **Motor speed regression** | | | | | | |
| Metabolite | 3.97 | -0.17, 8.11 | 0.06 | 3.35 | -0.86, 7.57 | 0.12 |
| Group | 0.14 | -6.06, 6.33 | 0.97 | 0.12 | -6.10, 6.34 | 0.97 |
| Metabolite x Group | -2.44 | -9.15, 4.27 | 0.47 | -0.88 | -7.65, 5.88 | 0.80 |
| **Executive function regression** | | | | | | |
| Metabolite | 0.09 | -1.15, 1.33 | 0.88 | 0.50 | -0.75, 1.74 | 0.43 |
| Group | 0.40 | -1.42, 2.22 | 0.66 | 0.32 | -1.48, 2.11 | 0.73 |
| Metabolite x Group | 1.17 | -0.74, 3.07 | 0.23 | 0.96 | -0.92, 2.85 | 0.31 |
| **Global cognition (BACS-t) regression** | | | | | | |
| Metabolite | 3.00 | -1.01, 7.01 | 0.14 | 3.58 | -0.48, 7.64 | 0.08 |
| Group | 0.75 | -5.92, 6.79 | 0.81 | 1.03 | -5.00, 7.07 | 0.73 |
| Metabolite x Group | -0.13 | -6.69, 6.44 | 0.97 | -2.31 | -8.93, 4.30 | 0.49 |
| **Global cognition (BACS-z) regression** | | | | | | |
| Metabolite | 0.32 | -0.09, 0.72 | 0.12 | 0.37 | -0.04, 0.78 | 0.07 |
| Group | 0.13 | -0.48, 0.73 | 0.68 | 0.15 | -0.45, 0.75 | 0.62 |
| Metabolite x Group | -0.09 | -0.75, 0.56 | 0.78 | -0.31 | -0.96, 0.34 | 0.35 |

**Figure S4. Interaction Plots. Predictive margins of TRS with 95% confidence intervals. Anterior Cingulate Cortex glutamate is the independent variable of interest.**


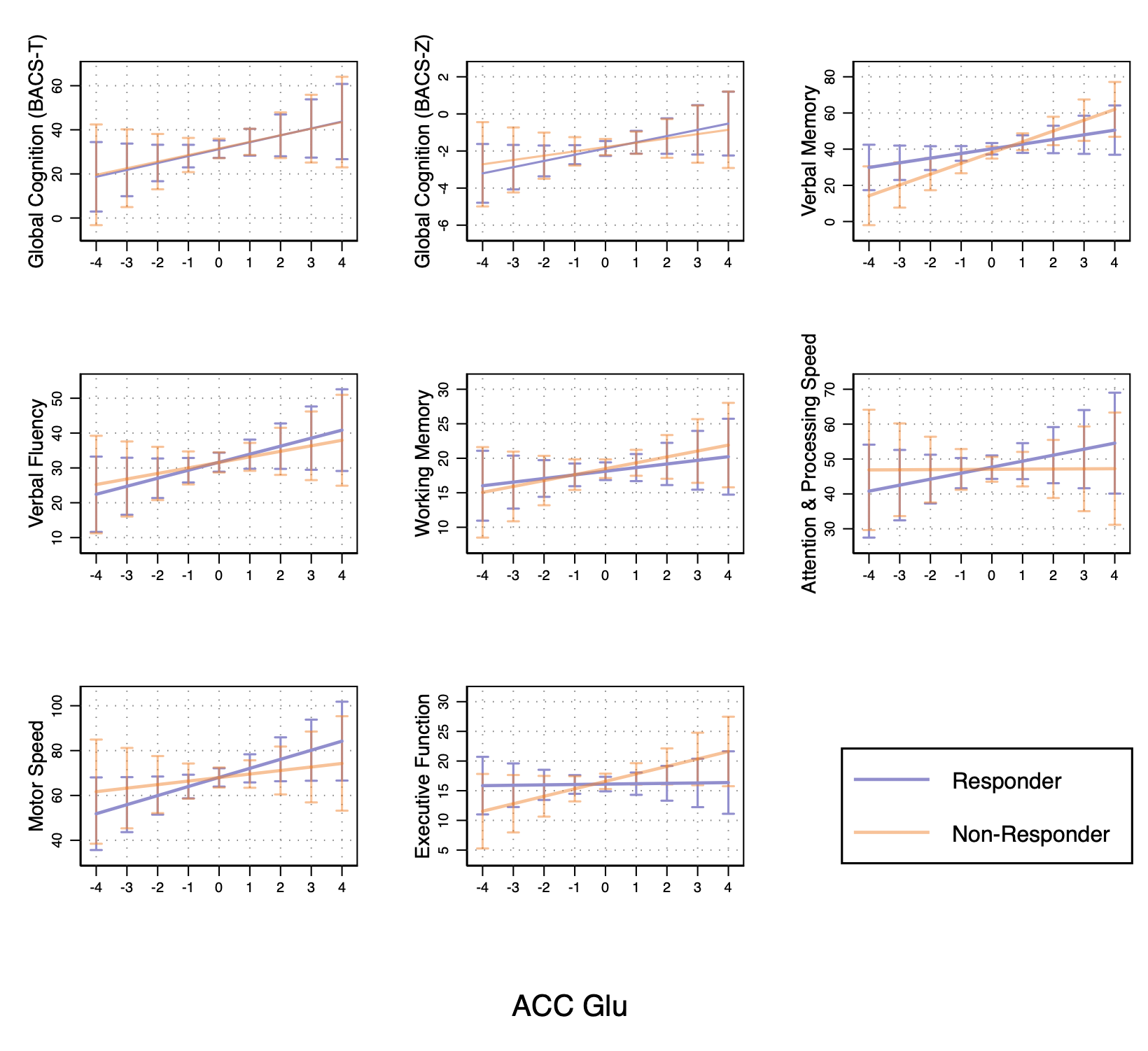


**Figure S5. Interaction Plots. Predictive margins of TRS with 95% confidence intervals. Anterior Cingulate Cortex Glx is the independent variable of interest.**


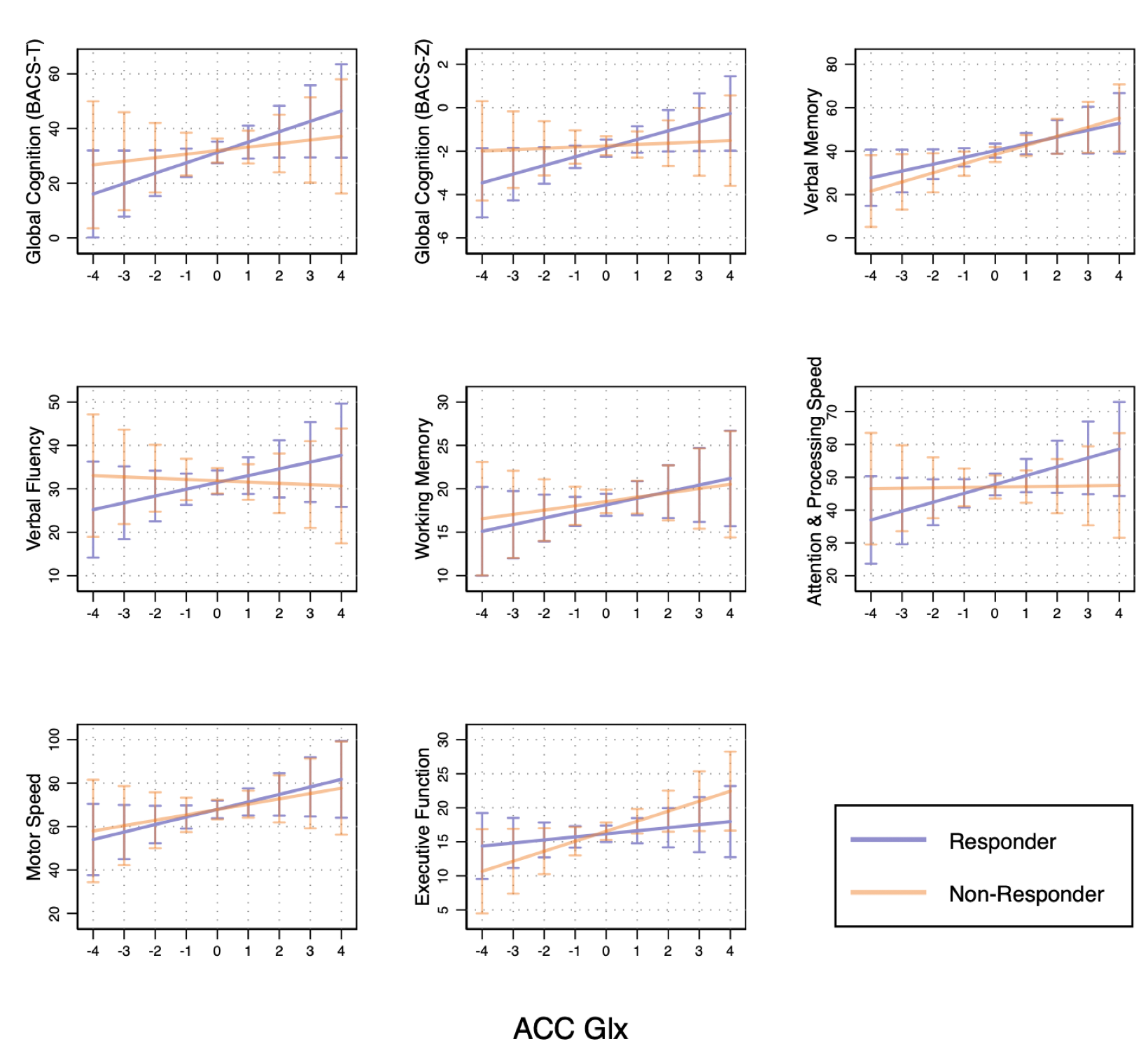

Supplement: Supplementary file 1 — Supplementary Materials. Impaired verbal memory function is related to anterior cingulate glutamate levels in schizophrenia: findings from the STRATA study [file 41537_2022_265_MOESM1_ESM.docx]
